# The current riboswitch landscape in Clostridioides difficile

**DOI:** 10.1099/mic.0.001508

**Published:** 2024-10-15

**Authors:** Adriana Badilla Lobo, Olga Soutourina, Johann Peltier

**Affiliations:** 1 Université Paris-Saclay, CEA, CNRS, Institute for Integrative Biology of the Cell (I2BC), 91198, Gif-sur-Yvette, France

**Keywords:** *C. difficile*, pathogen, riboswitch, therapeutics

## Abstract

Riboswitches are 5′ RNA regulatory elements that are capable of binding to various ligands, such as small metabolites, ions and tRNAs, leading to conformational changes and affecting gene transcription or translation. They are widespread in bacteria and frequently control genes that are essential for the survival or virulence of major pathogens. As a result, they represent promising targets for the development of new antimicrobial treatments. *Clostridioides difficile*, a leading cause of antibiotic-associated nosocomial diarrhoea in adults, possesses numerous riboswitches in its genome. Accumulating knowledge of riboswitch-based regulatory mechanisms provides insights into the potential therapeutic targets for treating *C. difficile* infections. This review offers an in-depth examination of the current state of knowledge regarding riboswitch-mediated regulation in *C. difficile*, highlighting their importance in bacterial adaptability and pathogenicity. Particular attention is given to the ligand specificity and function of known riboswitches in this bacterium. The review also discusses the recent progress that has been made in the development of riboswitch-targeting compounds as potential treatments for *C. difficile* infections. Future research directions are proposed, emphasizing the need for detailed structural and functional analyses of riboswitches to fully harness their regulatory capabilities for developing new antimicrobial strategies.

## Introduction

**Fig. 1.**
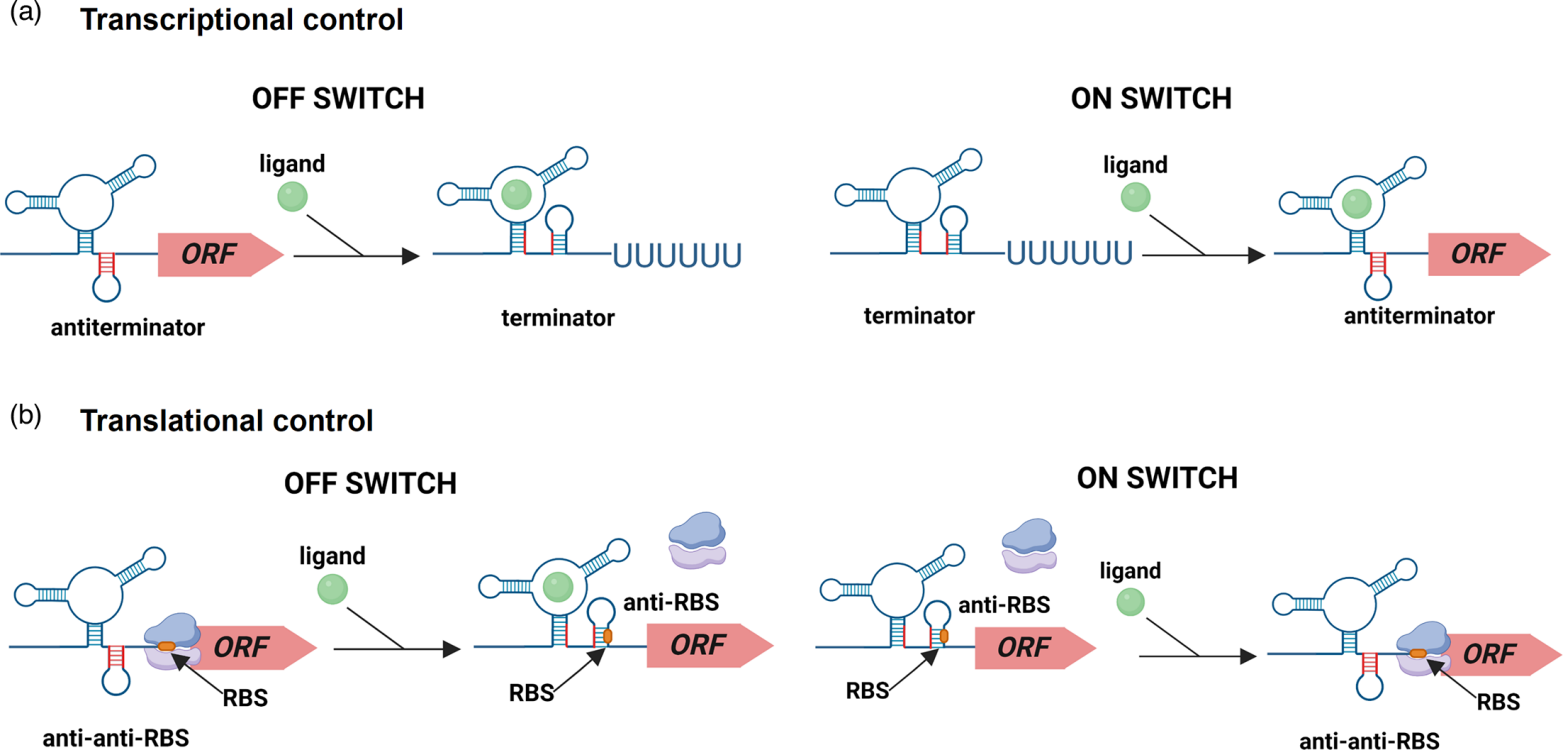
General mechanism of riboswitch action at the transcriptional or translational levels. (a) Regulation mechanism of riboswitches controlling the transcription termination. The binding of the ligand to the riboswitch aptamer induces a conformational change. In the case of an ‘OFF’ switch, it results in the formation of a terminator triggering the premature termination of the transcription and preventing the gene expression. In the case of an ‘ON’ switch, an antiterminator is formed, leading to the transcription of the downstream ORF. (**b)** Regulation mechanism of riboswitches controlling the translation initiation. Ligand binding induces a conformational change resulting either in the sequestration of the RBS, which prevents the translation (‘OFF’ switch), or in the formation of an anti-anti-sequestrator structure releasing the RBS to allow ribosome binding and translation. Figure created with BioRender.com.

Pathogenic bacteria are under constant pressure from environmental stressors, including nutrient and metabolite deficiencies. To survive and timely express virulence genes, they must quickly detect, respond to and adapt to the changes in their environment. Together with two-component systems and alternative sigma factors, known as key players in regulating gene expression in response to stresses [1], non-coding RNA (ncRNA) molecules or regulatory RNAs largely contribute to the adaptive responses and the regulation of metabolic processes [2]. Riboswitches, a type of *cis*-acting RNAs, are found in the 5′-untranslated region (5′-UTR) of mRNA and usually contain two distinct functional domains [3]. The aptamer domain is a conserved and highly folded structured RNA element that acts as a sensor, selectively recognizing a cognate small molecule [4]. The binding of the ligand to the aptamer domain triggers a conformational change in the downstream expression platform, which is a highly variable region [5]. This alternative folding of the expression platform negatively or positively modulates the gene expression primarily through intrinsic termination of transcription and prevention or activation of translation, by forming a transcriptional terminator or RBS sequestrator secondary structure, or stabilizing an antiterminator or antisequestrator structure [6] (Fig. 1). Intriguingly, Gram-positive bacteria preferentially use a riboswitch aptamer domain associated with a transcriptional expression platform, while Gram-negative bacteria favour translational riboswitch-mediated regulations [7,8]. A number of other mechanisms, albeit rarer, including alteration of mRNA stability and alternative self-splicing, have also been described, and their regulatory details have been extensively reviewed elsewhere [4,9, 10]. While the majority of riboswitches act as ‘OFF’ switches, turning off the expression of downstream genes in response to ligand binding, ‘ON’ switches have also been characterized [9].

Riboswitches are a widespread form of regulation in bacteria, having evolved to bind a diverse array of ligands, including coenzymes, amino acids, nucleotide derivatives, ions and other metabolites [3]. An additional class of riboswitches, known as T-box riboswitches, is unique in that it binds to tRNA molecules rather than small-molecule ligands [11]. To date, the function of more than 55 classes of riboswitches has been validated, but thousands more are predicted to exist [3,12].

*Clostridioides difficile*, a major cause of antibiotic-associated nosocomial diarrhoea in adults, harbours a significant number of ncRNAs and largely uses riboswitches to control the gene expression [13,17]. This underscores the importance of RNA-based gene regulation in this bacterium. *C. difficile* is a strictly anaerobic, spore-forming Gram-positive bacterium, and its transmission is mediated by the contamination of the gut by spores. The germination of *C. difficile* spores in the digestive system is triggered by antibiotic-induced microbiome dysbiosis, leading to the multiplication of *C. difficile* vegetative cells and colonization of the intestine [18]. The main virulence factors of the bacterium are two secreted toxins, TcdA and TcdB, which target the gut epithelium, causing severe inflammation and damage to the colon [19]. *C. difficile* is a significant antimicrobial-resistant pathogen whose infections are associated with high recurrence rates, and an imperative is to define alternative treatment strategies as well as new antibiotic targets. Riboswitches are attractive targets for new drugs because they are almost exclusively found in bacteria and often control the expression of genes essential for survival or pathogenesis [20]. In this review, we will describe the different riboswitch classes found in *C. difficile*, the pathways they regulate and the developments to use them as targets of novel therapeutics.

## Overview of riboswitches in *C. difficile*

**Fig. 2.**
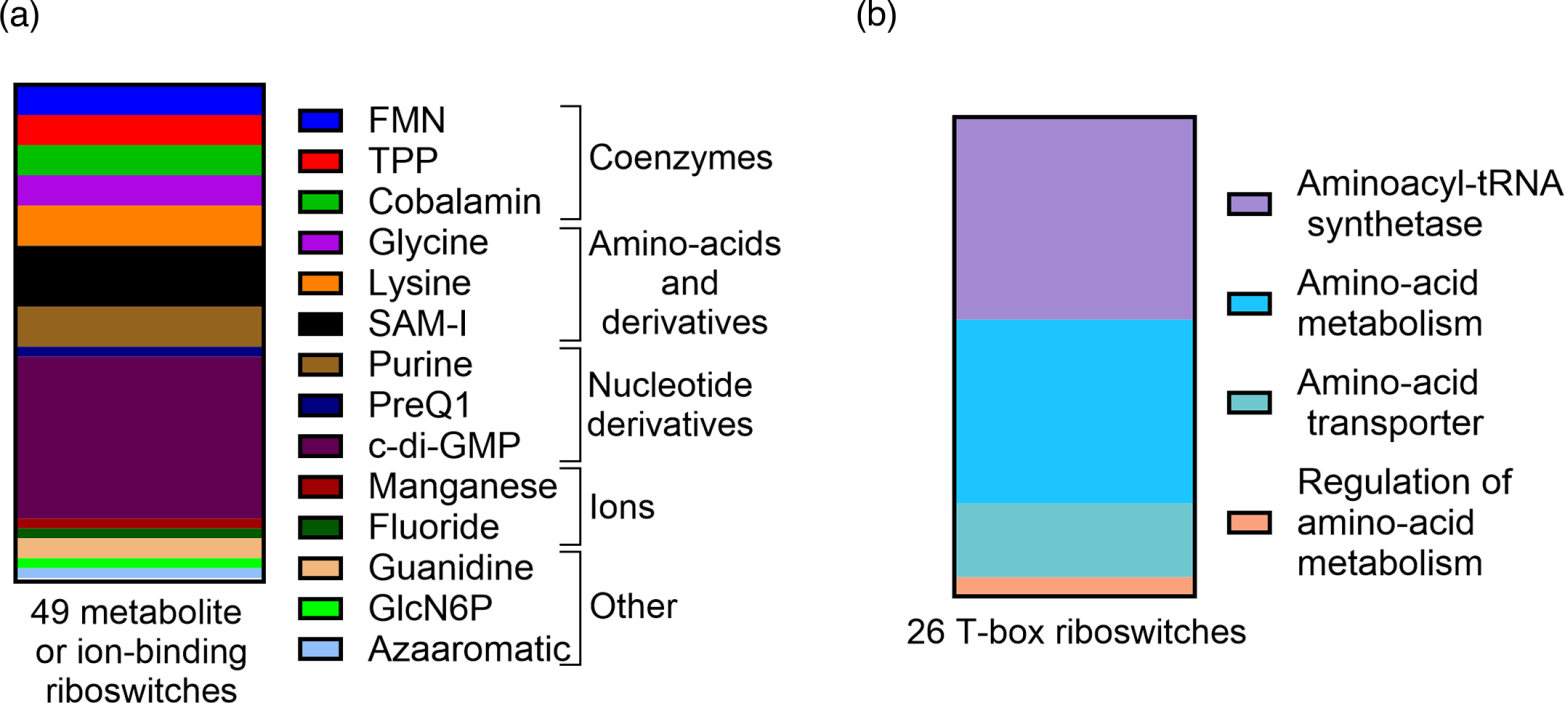
Diversity of riboswitches in *C. difficile*. (a) Forty-nine metabolites or ion-binding riboswitches have been identified in the genome of *C. difficile* so far. The different riboswitch ligands and the biochemical category they belong to are indicated. (**b)** *C. difficile* harbours 26 T-box riboswitches. The functional categories of the genes downstream the T-box riboswitches are indicated.

The arsenal of riboswitches identified by the combination of transcriptomic and bioinformatic analyses in *C. difficile* 630Δ*erm*, a commonly used spontaneous erythromycin-sensitive derivative of the historical strain 630, includes 49 Rfam-predicted metabolite or ion-binding riboswitches (Fig. 2a, Table 1) and 26 T-box riboswitches (Fig. 2b, Table 2) [13,17,21].

**Table 1.** Riboswitches binding small-molecule ligands and riboswitch candidates in *C. difficile*

Riboswitch class or RNA motif	Downstream CDS or operon	Associated function
FMN	*CD0147* (*ribU*)	Riboflavin transporter
	*CD1700-1697* (*ribD*, *E*, *BA*, *H*)	Riboflavin biosynthesis
	*CD3299*	Drug efflux protein of the EmrB/QacA subfamily
TPP	*CD1599-1601* (*thiD*, *thiM*, *thiE*)	TPP metabolism
	*CD1702-06* (*thiC*, *S*, *F*, *G*, *H*, *E2*)	TPP metabolism
	*CD1980.1-1978*	ATP binding cassette (ABC)-type transport system
Cobalamin	*CD0324-7* (*cbiM*, *N*, *Q*, *O*)	ECF-type transporter involved in cobalt import
	*CD2999-7*	ABC-type transport system
	*CD3435-19* (*cob* and *cbi* genes)	Cobalamin biosynthesis
Glycine	*CD1657-58* (*gcv* operon)	Glycine cleavage system
	*CD2276*	Sodium:alanine symporter
	*CD2357-48* (*grd* operon)	Glycine fermentation through Stickland reactions
Lysine	*CD1655*	Na^+^/H^+^ antiporter NhaC like
	*CD2054-53* (*lysC*, *A*)	Lysine biosynthesis
	*CD3100-3099* (*yfcc*)	Basic amino acid antiporter; aminohydrolase
	*CD3224-3227* (*asd*, *dapA3*, *dapB1*, *dapH*)	Lysine biosynthesis
SAM-I	*CD0130* (*metK*)	S-Adenosylmethionine synthetase
	*CD1166* (*hom1*)	Homoserine dehydrogenase
	*CD1489-91* (*metN*, *L*, *Q*)	Methionine-specific ABC-type transport system
	*CD1653*	d-Methionine-binding lipoprotein
	*CD1825-26* (*metY, A*)	O-Acetylhomoserine sulfhydrylase, homoserine O-succinyltransferase
	*CD3598* (*luxS*)*-CD3596*	Methionine biosynthesis, includes LuxS
Purine	*CD0198* (*guaA*)*-0198.1*	Glutamine-hydrolysing guanosine monophosphate (GMP) synthase; hypothetical protein
	*CD2107*	Xanthine/uracil/vitamin C permease of AgzA family
	*CD2704*	Xanthine/uracil/vitamin C permease of AgzA family
	*CD2330* (*xpt*)	Xanthine phosphoribosyltransferase
PreQ1	*CD1682*, *84* (*queK*, *qrtT*, *queL*)	PreQ1 salvage
c-di-GMP-I	*CD0245-0272* (early-stage flagellar operon)	Flagellar biosynthesis
	*CD1424*	Small hypothetical protein
	*CD1981.2*	Small hypothetical protein
	*CD1990*	SH3 domain protein
	*CD1990.3*	Small hypothetical protein
	*CD2309*	Small hypothetical protein
	*CD2797*	Calcium-binding adhesion protein
	*CD2830* (*ppep-1*)	Metalloprotease
	*CD3368.2*	Small hypothetical protein
	RCd11 antisense RNA	Antitoxin of type I toxin/antitoxin system
	RCd12 antisense RNA	Antitoxin of type I toxin/antitoxin system
	Orphan riboswitch between *CD2517* and *CD2517.1*	No known function
c-di-GMP-II	*CD2831*	Collagen-binding protein
	*CD3246*	Thioester domain protein
	*CD3267-65* (*cmrR*, *S*, *T*)	Signal transduction system
	*CD3513-03* (pilin operon)	Type IV pilus synthesis
Manganese	*CD2503*	Cation-translocating P-type ATPase
Fluoride	*CD0656-7*	ClC-type ion channel protein; NhaP-type Na^+^/H^+^ or K^+^/H^+^ antiporter
Guanidine-I	*CD0488* (*gdx*)	Guanidine exporter
	*CD2392* (*cdtA*)	Deacylation of canavanylated tRNA^Arg^
glmS	*CD0120* (*glmS*)	Glucosamine-fructose-6-phosphate aminotransferase
yjdF	*CD1762*	YjdF family protein
uup	*CD3359*	ATPase component of ABC transporters
bglG	*CD3117-5* (*bglG*, *F*, *A*)	β-Glucoside phosphotransferase system
raiA	*C0134-8*	Phosphotransferase system (PTS), lactose/cellobiose family (ComFC-like protein encoded by *CD0133* upstream of the raiA motif)
speF	*CD1759*	Methyltransferase domain protein

**Table 2.** T-box riboswitches identified in *C. difficile*

Functional class regulated by the T-box riboswitch	CDS downstream the T-box riboswitch	Associated function
Aminoacyl-tRNA synthetase	*CD0014*	Serine-tRNA ligase
	*CD0049*	Proline-tRNA ligase
	*CD0574*	Threonine-tRNA ligase
	*CD0699*	Phenylalanyl-tRNA synthetase alpha chain
	*CD1282*	Alanine-tRNA synthetase
	*CD1521*	Tyrosine-tRNA ligase
	*CD2245*	Asparagine-tRNA ligase
	*CD2521*	Leucine-tRNA ligase
	*CD2618*	Isoleucine-tRNA ligase
	*CD3256*	Valine-tRNA ligase
	*CD3540*	Methionine-tRNA ligase
Amino acid metabolism	*CD0989*	2-Isopropylmalate synthase
	*CD1580*	Homoserine dehydrogenase
	CD1580*	Homoserine dehydrogenase
	*CD1785*	Argininosuccinate synthase
	*CD2034*	*N*-Acetyl-gamma-glutamyl-phosphate reductase
	*CD2118*	Threonine synthase
	*CD2500*	Argininosuccinate lyase
	*CD2611*	Bifunctional 5′-methylthioadenosine/S-adenosylhomocysteine nucleosidase
	*CD2695*	Aspartate-ammonia ligase
Amino acid transporter	*CD0750*	ATP binding cassette (ATP)-type transport system, aminoacid-family extracellular solute-binding protein
	*CD1716*	Putative cytosine permease
	*CD1774-1776*	ABC-type transport system, aminoacid-family extracellular solute-binding protein
	*CD1988*	Putative tryptophan transport protein
Regulation of amino acid metabolism	*CD1564*	ACT (aspartate kinase-chorismatemutase-tyrA) domain-containing protein

1 *Two distinct T-boxes precede *CD1580*.

## Coenzyme-binding riboswitches

The largest category of riboswitch ligands is coenzymes. This family of riboswitches is diverse, and some members, including the thiamine pyrophosphate (TPP), cobalamin and FMN riboswitches, are very common in bacteria and present in *C. difficile* (Fig. 2a, Table 1). Other riboswitches responding to the molybdenum and tungsten cofactor (Moco and Tuco) [22], the biologically active form of folate tetrahydrofolate (THF) [23,24], the major byproduct of S-adenosylmethionine (SAM)-mediated methylation reactions S-adenosyl-l-homocysteine [25] or the ubiquitous enzyme cofactor nicotinamide adenine dinucleotide (NAD^+^) [26] are less widely represented and are not found in *C. difficile*.

### FMN riboswitches

FMN riboswitches are widely found in bacterial species and regulate the expression of genes involved in the biosynthesis and transport of riboflavin (vitamin B_2_) and related compounds [27]. FMN is a derivative of riboflavin and is required as a cofactor for several flavoprotein-mediated redox reactions [28]. Three FMN riboswitches are predicted in the genome of *C. difficile*. One FMN riboswitch is located in the 5′-UTR of the riboflavin biosynthesis operon, which includes *ribD*, *ribE*, *ribBA* and *ribH*. The *ribD* FMN riboswitch operates through transcription termination regulation in *Bacillus subtilis* [27]. Another FMN riboswitch is found upstream of a *ribU* orthologue, which encodes a riboflavin transporter in *B. subtilis* [29]. The associated FMN riboswitch controls the translation initiation of RibU in *B. subtilis* [27]. Additionally, *C. difficile*’s genome harbours a subtype 2 variant FMN riboswitch that is only present in *Clostridium* and *Atopobium* species, as well as in some bacterial metagenomic DNA sequences [30]. In *C. difficile*, this variant is located upstream of a gene encoding a putative drug efflux protein of the EmrB/QacA subfamily that belongs to the major facilitator superfamily. This protein was initially reported to function as a riboflavin importer [31], but several lines of evidence suggest that it is likely not its main function. First, this FMN riboswitch variant was shown to function as an ‘ON’ switch regulating the transcription termination, which is the opposite of what one would expect for a riboflavin transporter. Second, *C. difficile* already possesses the riboflavin transporter RibU. Finally, the subtype 2 variant FMN riboswitch exhibits nucleotide differences in the binding pocket compared to genuine FMN riboswitches and does not bind FMN. It binds instead to toxic photochemical breakdown products of FMN such as lumichrome and lumiflavin [30]. These data suggest the potential involvement of the variant FMN riboswitch in the activation of efflux pump production as a detoxification pathway of its cognate ligand.

FMN riboswitches constitute promising targets for therapeutic development. The natural riboflavin analogue roseoflavin produced by *Streptomyces davawensis* was shown to be phosphorylated inside the cells to bind the FMN riboswitch and inhibit the growth of *B. subtilis* and *Listeria monocytogenes* [32,34]. Roseoflavin-resistant strains with mutations in the FMN riboswitch, which resulted in the overproduction of riboflavin, could be isolated, showing that the riboswitch is the primary target of the analogue [34,36].

Among FMN analogues, the synthetic fluoro-phenyl analogue of FMN, 5FDQD (5-(3-(4-fluorophenyl)butyl)−7,8-dimethylpyrido(3,4-*b*)quinoxaline-1,3(2*H*,5*H*)-dione), demonstrated a potent and highly selective antibacterial activity against *C. difficile in vitro* and *in vivo* in a mouse model, preserving the caecal microbiota [37,38]. Designed to be cell permeable, 5FDQD has been shown to bind to and trigger the conformational change of the *ribD* FMN riboswitch of *B. subtilis* with the same potency as FMN. Additionally, the presence of fluorine in 5FDQD has recently been shown to enhance the binding affinity of the analogue to the FMN riboswitch [39]. This suggests that the antibacterial activity of 5FQD is triggered by binding to the FMN riboswitches of *C. difficile*, although the precise mechanism of action remains to be determined. As an alternative to FMN analogues, chimeric antisense oligonucleotides complementary to the aptamer sequence of the FMN riboswitch have been engineered [40]. The formed double-stranded RNA/DNA hybrid was enzymatically digested by the endonuclease RNase H, leading to mRNA degradation. These antisense oligonucleotides, covalently bound to the vascular endothelial-cadherin-derived peptide (pVEC), a cell-penetrating peptide of 18 aas [41], were shown to inhibit the growth of *E. coli*, *L. monocytogenes* and *Staphylococcus aureus* [40]. A modified antisense oligonucleotide with eight mismatches did not affect the growth of the different bacteria, suggesting that the growth defect is caused by the specific interaction between the oligonucleotide and the FMN riboswitch.

### TPP riboswitches

TPP riboswitches are the most widespread riboswitch class known in bacteria and the only class of riboswitches also identified in eukaryotes, including plants, fungi and algae [42,44]. In bacteria, they control the gene expression through transcription termination or prevention of translation [6]. TPP riboswitches regulate the operons involved in the synthesis and transport of thiamine (vitamin B_1_) and its phosphorylated derivatives, including the biologically active form of thiamine, TPP. TPP is an essential coenzyme in carbohydrate metabolism, including glycolysis, the citric acid cycle and the pentose phosphate pathway [45]. Three TPP riboswitches are predicted in *C. difficile*, two of which control genes involved in TPP metabolism (*thiD-M-E* and *thiC-S-F-G-H-E2*) and the third is located upstream of an operon encoding an ATP binding cassette (ABC)-type transport system. It is possible that this transporter would import thiamine into the cell even though it shows no significant homologies to thiamine transporters described in other bacteria.

TPP analogues binding to the TPP riboswitches may represent promising drugs to tackle bacterial infections. As a proof of principle, pyrithiamine pyrophosphate, the phosphorylated form of the thiamine analogue pyrithiamine, was shown to bind TPP riboswitches and turn off the expression of downstream genes in *B. subtilis* [46]. The toxicity of pyrithiamine in bacteria and fungi is probably at least in part mediated by the binding of pyrithiamine pyrophosphate to TPP riboswitches, which results in TPP starvation for cells. TPP analogues binding to the TPP riboswitches and altering downstream gene expression *in vitro* have since then been developed [47,48]. In addition, an antisense oligonucleotide bound to the cell-penetrating peptide pVEC was shown to bind the TPP riboswitch and inhibit downstream gene expression [49]. The oligonucleotide was shown to inhibit the growth of *L. monocytogenes* and *B. subtilis*.

### Cobalamin riboswitches

Cobalamin riboswitches, also known as B_12_ riboswitches or adenosylcobalamin (AdoCbl) riboswitches, are among the most widespread and structurally diverse riboswitch types in bacteria [3]. They respond to different forms of cobalamin and function as ‘OFF’ switches to regulate the transport or biosynthesis of this important cofactor at the transcriptional or translational level [50,52]. Cobalamin is essential for the activity of several important enzymes involved in a variety of transmethylation and rearrangement reactions [53]. The two most biologically active forms of cobalamin are AdoCbl and methylcobalamin (MeCbl), while inactive forms include cyanocobalamin (vitamin B12), hydroxocobalamin (HyCbl) and aquocobalamin [54]. The large majority of cobalamin riboswitches are class I riboswitches specifically responding to AdoCbl. However, two minor groups of variants with a different architecture, referred to as class IIa and class IIb riboswitches, have been reported [55]. Class IIa riboswitches are found in cyanobacteria and bacterial metagenomes and are highly selective for MeCbl and HyCbl over AdoCbl. Class IIb riboswitches are associated with ethanolamine utilization genes in *Enterococcus faecalis* and *L. monocytogenes* and interact highly selectively with AdoCbl. Three class I cobalamin riboswitches are found in the genome of *C. difficile* upstream of a 17-gene operon-encoding cobalamin biosynthesis enzymes and two operons of 4- and 3-gene coding, respectively, for an energy-coupling factor (ECF)-type transporter involved in cobalt import and an ABC-type transport system homologue to the cobalamin uptake system BtuCDF of *E. coli* [56]. Targeting these riboswitches could lead to the deprivation of cobalamin and the inhibition of bacterial growth. However, diverse cobalamin derivatives were recently shown to bind to class II but not to class I riboswitches, revealing that the latter strongly discriminate between the natural AdoCbl ligand and analogues [57].

## Riboswitches binding amino acids and their derivatives

Metabolite-binding riboswitches play a crucial role in controlling amino acid availability in bacteria. For instance, glycine riboswitches regulate the expression of genes involved in glycine metabolism and transport by binding to glycine [58,59]. Similarly, lysine riboswitches modulate the metabolism of lysine and its precursors, which are common to threonine and methionine, through interactions with lysine. In addition, cyanobacteria and marine metagenomes have been found to possess glutamine-sensing riboswitches that regulate nitrogen metabolism [60]. Methionine and cysteine biosynthesis, on the other hand, is regulated by the SAM-I riboswitch (also named S-box riboswitch), which responds to SAM binding. SAM is the main methyl donor for transmethylation reactions in all living organisms and is synthesized from methionine and ATP by SAM synthetase [61,62]. In *C. difficile*, three glycine, four lysine and six SAM-I riboswitches have been identified [13,15] (Fig. 2a, Table 1).

### Glycine riboswitches

Glycine riboswitches in *C. difficile* control the expression of genes encoding the subunits A and B of a glycine cleavage (GCV) system, a glycine reductase (*grd*) operon and a gene encoding a putative alanine or glycine:cation symporter (AGCS). High intracellular glycine concentrations are toxic to bacterial physiology, as they interfere with peptidoglycan synthesis [63]. This interference occurs because glycine can be incorporated in place of alanine into peptidoglycan precursors, but these modified precursors serve as poor substrates for the peptidoglycan synthesis enzymes. Therefore, glycine catabolism is essential to maintain appropriate levels within cells and ensure efficient bacterial growth. The GCV system is a major pathway for glycine degradation and catalyses the oxidative cleavage of glycine to CO_2_, NH^4+^ and a methylene group, which is accepted by THF to form 5,10-methylene-THF. The glycine riboswitch located upstream of the GCV-encoding genes functions as an ON switch, activating the gene transcription under high glycine conditions. This system has been shown to be important for glycine detoxification in *B. subtilis* and *Streptomyces griseus* [59,64, 65].

The Grd reductive pathway, which is primarily found in species of *Clostridia*, catabolizes glycine through substrate-level phosphorylation to generate energy. Such pathways for amino acid fermentation are known as Stickland reactions, which involve the coupled oxidation and reduction of amino acids for energy generation [66,67]. The glycine fermentation pathway, encoded by an eight-gene operon in *C. difficile*, is an important metabolic pathway promoting sporulation and pathogenesis, although further investigation is needed to determine what links these processes to glycine reduction [68]. The addition of glycine in a *C. difficile* minimal medium increased the transcription of the *grd* operon [68], suggesting that the glycine riboswitch located upstream of the operon operates as an ‘ON’ switch.

The members of the AGCS family transport alanine and/or glycine in symport with Na^+^ and or H^+^ and are often associated with glycine riboswitches [69,70]. Only a few members of this transporter family have been characterized. The Cyc system in *E. coli* transports glycine, d-alanine and d-serine, while an AGCS transporter of the halotolerant cyanobacterium *Aphanothece halophitica* takes up glycine and glutamine [71,72]. In contrast to the riboswitches located upstream of glycine catabolism pathways, glycine riboswitches located upstream of an AGCS transporter in *Vibrio cholerae* and *Streptococcus pyogenes* have been shown to function as OFF switches, with the gene expression being repressed by the presence of glycine [73,74]. This suggests that the glycine riboswitch upstream of the AGCS of *C. difficile* inhibits the synthesis of the glycine transporter to limit the glycine uptake when it is present in excess in the extracellular environment.

Because glycine riboswitches do not regulate the production of essential metabolites, they have not been exploited as antibacterial drug targets to date [75].

### Lysine riboswitches

Riboswitches that respond to the intracellular concentration of lysine, known as l-boxes, regulate the expression of two operons involved in lysine biosynthesis in *C. difficile*. This organization is common in all prokaryotes, and such riboswitches have been demonstrated to function as ‘OFF’ switches in *B. subtilis* and *E. coli* [76,77]. A lysine riboswitch is also present upstream of a gene belonging to the NhaC Na^+^/H^+^ antiporter superfamily. This genetic organization is present in several organisms, but the function of NhaC remains unknown [78]. It has been speculated that NhaC could potentially serve as a lysine transporter, but it may also function as a genuine Na^+^/H^+^ antiporter that regulates cellular pH when intracellular levels of lysine are low [78,79]. In the presence of high lysine concentrations, lysine decarboxylase, which consumes a proton upon conversion of l-lysine into cadaverine, would play this role instead. The fourth lysine riboswitch found in *C. difficile* is located upstream of a gene encoding the putative basic amino acid antiporter YfcC. Amino acid antiporters mediate the exchange of groups of amino acids, but the function of YfcC remains to be characterized. In *C. difficile*, *yfcC* is in apparent operon structure with a gene encoding a putative amidohydrolase of the M20D family.

Lysine biosynthesis is an essential metabolic pathway making the lysine riboswitch a valuable drug target. Several lysine analogues, including l-aminoethylcysteine (AEC), that inhibit the growth of *B. subtilis* have been developed [79,80]. These analogues bind to the *lysC* riboswitch from *B. subtilis* and repress riboswitch-mediated reporter gene expression. However, AEC also binds to lysyl-tRNA synthetase (LysRS) and is therefore incorporated into proteins, leading to the observed toxicity [81]. LysRS variants with reduced affinity for AEC conferred resistance to this compound, revealing that the interaction with the *lysC* riboswitch was not sufficient to inhibit growth [81].

### SAM-I riboswitches

The SAM-I riboswitch only precedes genes and operons involved in the metabolism and transport of methionine and SAM in *C. difficile* (Table 1). It is found upstream of the *hom1* gene, the *metY-metA* operon and a second operon of three genes comprising *luxS*, all involved in methionine synthesis. It is also located upstream of the stand-alone gene *metK*, which encodes the SAM synthetase, converting l-methionine into SAM. LuxS is involved in the detoxification of S-adenosylhomocysteine generated from SAM during the activated methyl cycle and creates 4,5-dihydroxy-2,3-pentanedione (DPD) as a by-product [82]. DPD is unstable and spontaneously cyclizes into different forms collectively known as autoinducer-2 (AI2). AI2 acts as a quorum-sensing signalling molecule and induces the expression of virulence genes and biofilm formation in many bacteria including *C. difficile* [82,83]. The SAM-I riboswitch is also located upstream of the *metN-metL-metQ* operon, which encodes a putative methionine-specific ABC-type transporter system. A gene encoding a MetQ paralog, putatively involved in methionine binding and also associated with a SAM-I riboswitch, is present in the genome of *C. difficile*. SAM-I is involved in a negative feedback regulatory mechanism to repress methionine biosynthesis in response to increasing SAM concentrations [62].

Although the inhibition of biosynthesis of the essential metabolites methionine and SAM has the potential to inhibit bacterial growth, SAM analogues targeting SAM riboswitches have been underexploited. Using virtual screening approaches, a few molecules with predicted binding capacity to a SAM-I riboswitch from *B. subtilis* were very recently identified [84]. However, their binding affinity to the riboswitch *in vitro* and their antibacterial activity remain to be investigated. Antisense oligonucleotides covalently bound to the cell-penetrating peptide pVEC and targeting the SAM-I riboswitch have also been engineered [85]. These antisense oligonucleotides were shown to inhibit the growth of *L. monocytogenes* and *S. aureus* [85].

### Riboswitches binding nucleotide derivatives

Riboswitches binding purines and derivative compounds are highly prevalent in bacteria. The purine riboswitch family is comprised of the guanine, adenine, xanthine and 2′-deoxyguanosine riboswitch classes, which control the purine biosynthesis and transport in bacteria [86,87]. The guanine and adenine riboswitches are structurally similar, and their ligand specificity arises from the formation of a Watson–Crick bp with nucleotide 74, which is always a cytosine in the guanine class and a uridine in the adenine class [88]. The 2′-deoxyguanosine and xanthine classes share a global architecture similar to the other purine riboswitches but exhibit differences in the ligand-binding pocket [87,89]. Purine derivative-binding riboswitches comprise members binding prequeuosine-1 (PreQ_1_) [90], a 7-aminomethyl-7-deazaguanine metabolite precursor of the hypermodified guanine nucleotide queuosine (Q) and purine-based signalling molecules. Signalling molecules detected by specific riboswitches include the cyclic dinucleotides cyclic di-GMP (c-di-GMP) [91,92], cyclic di-AMP (c-di-AMP) [93] and cyclic GMP-AMP [94,95], as well as the alarmones guanosine tetraphosphate (ppGpp) [96] and the modified purine biosynthetic intermediate 5-amino-4-imidazole carboxamide riboside 5′-triphosphate, also known as the Z nucleoside triphophate [97].

### Purine riboswitches

**Fig. 3.**
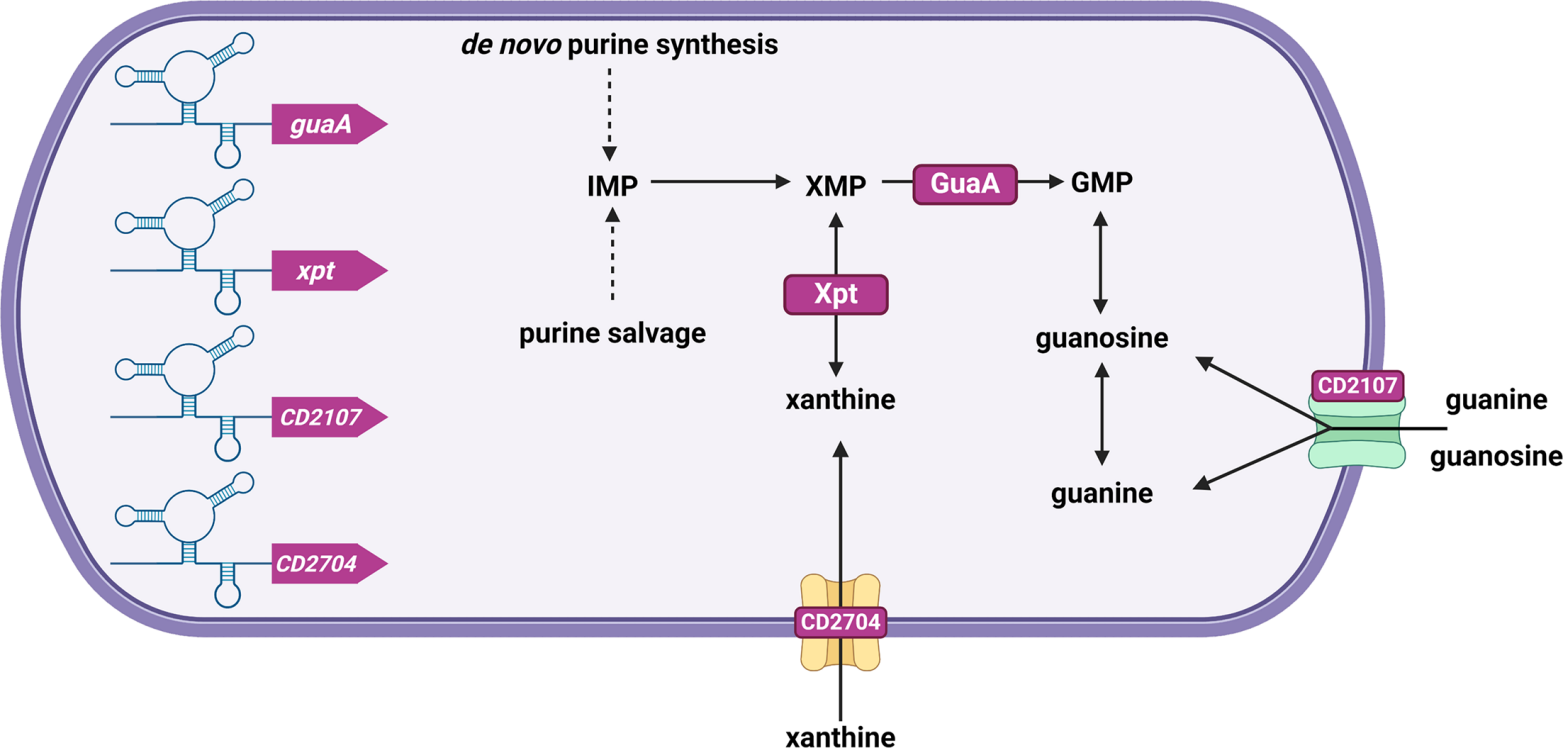
Control of the purine metabolism by riboswitches in *C. difficile*. Four guanine riboswitches acting as ‘OFF’ switches and controlling the expression of genes involved in the purine metabolism (*guaA* and *xpt*) or the transport of purine derivatives (*CD2107* and *CD2704*) are present in *C. difficile*. IMP, inosine 5′-monophosphate; XMP, xanthosine 5′-monophosphate; GMP, guanosine 5′-monophosphate. Figure created with BioRender.com.

The genome of *C. difficile* encodes four distinct purine riboswitches that bind guanine with high affinity [98], regulating the expression of genes involved in purine metabolism (Table 1). These riboswitches control the transcription of genes encoding two putative xanthine/uracil/vitamin C permeases of the AgzA family (CD2107 and CD2704), the xanthine phosphoribosyltransferase Xpt (XPRTase) and a two-gene operon encoding the glutamine-hydrolysing guanosine monophosphate (GMP) synthase GuaA and a small hypothetical protein of 41 aas. All four riboswitches turn off the gene expression when bound to their ligand [98]. The Xpt and GuaA riboswitches respond to guanine, xanthine and guanosine with different affinities. In contrast, the *CD2107* riboswitch preferentially responds to guanosine, and the *CD2704* riboswitch specifically responds to xanthine [98], suggesting that the two permeases are specialized in the respective transport of these two metabolites. Xpt and GuaA are involved in the conversion of precursor metabolites into GMP. GMP can be generated directly from guanine and guanosine but also from other purine analogues when the formers are not available. Xpt converts the purine analogue xanthine into xanthine monophosphate (XMP), which is, in turn, used by GuaA to produce GMP [99,100]. Alternatively, XMP can be generated from inosine monophosphate, synthesized either through the *de novo* purine synthesis pathway or from adenine through the purine salvage pathway [101] (Fig. 3).

The inactivation of *guaA* in *C. difficile* 630 Δ*erm* resulted in severe growth defects under nutrient-limiting growth conditions. Additionally, a *guaA* mutant displayed a significant reduction in the competitiveness and colonization capacity of *C. difficile* in a mouse model [98]. Notably, the pyrimidine analogue PC1 (2,5,6-triaminopyrimidine-4-one), which binds the only guanine riboswitch of *S. aureus* with a physiologically relevant affinity, inhibited the growth of several Gram-positive bacteria, including *C. difficile*. Only bacteria controlling the *guaA* expression through a guanine riboswitch responded to PC1, suggesting that growth inhibition was mediated by the riboswitch-mediated *guaA* repression [102]. These findings emphasize the importance of *de novo* GMP biosynthesis in *C. difficile* and highlight the *guaA* riboswitch as a promising target for the development of novel antibacterials. The purine analogue 6-*N*-hydroxylaminopurine (6-*N*-HAP) has also been shown to bind guanine riboswitches and inhibit downstream gene expression by inducing premature termination of the transcription in *B. subtilis* and *L. monocytogenes* [103,104]. This compound inhibited the growth of the two bacterial species. However, the antibacterial activity of 6-*N*-HAP in *L. monocytogenes* was shown to be independent of its interaction with the riboswitches and mediated by its mutagenic properties [104].

### PreQ1 riboswitches

The PreQ1 riboswitch downregulates the synthesis and transport of PreQ1, a precursor in the biosynthesis of Q, in response to its own binding [90,105]. Q is found in nearly all prokaryotes and eukaryotes in asparaginyl, aspartyl, histidyl and tyrosyl tRNAs at the anticodon wobble position [106]. It enhances the translational efficiency and fidelity likely by preventing ribosomal frameshifting [107]. PreQ1 riboswitches are grouped into three classes based on distinct sequence and structure conservation and include examples of both transcriptional and translational control mechanisms [90]. Whereas PreQ1-I riboswitches are widely distributed in the phyla Bacillota, Proteobacteria and Fusobacteria, the PreQ1-II and PreQ1-III riboswitches have been identified only in the order Lactobacillales and the family Oscillospiraceae of the Bacillota, respectively [90,108, 109]. A compound binding to the PreQ1 riboswitch of *B. subtilis* and modulating riboswitch transcriptional termination has been identified, but its antibacterial activity remains to be evaluated [110].

**Fig. 4.**
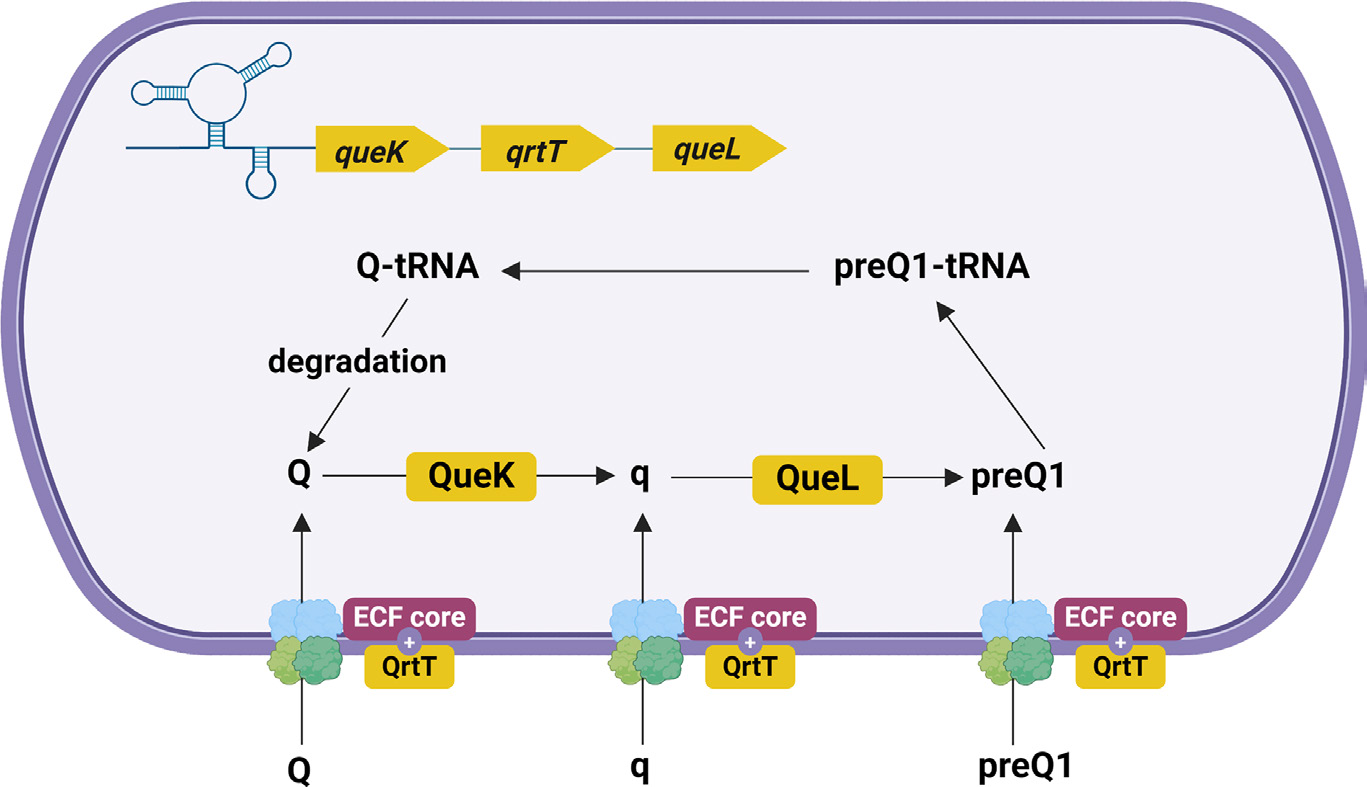
Control of the PreQ1 salvage pathway by riboswitches in *C. difficile*. A PreQ1-I riboswitch is located upstream of a three-gene operon-encoding QueK, QrtT and QueL, which are involved in PreQ1 salvage. QrtT is the substrate-specific component of a queuosine ECF transporter and interacts with the core components (ECF core) of this transporter. Figure created with BioRender.com.

*C. difficile* is unable to synthesize PreQ1 and relies on salvage pathways to acquire this metabolite [111]. A PreQ1-I riboswitch is located upstream of a three-gene operon encoding QueK, QrtT and QueL, which are involved in PreQ1 salvage in *C. difficile* (Fig. 4, Table 1). QrtT is a substrate-specific component of an ECF-type transporter that takes up PreQ_1_, Q and the queuine (q) base from the environment [111]. QueK and QueL are, respectively, a queuosine hydrolase that converts Q into q and a lyase that generates PreQ1 from q.

### C-di-GMP riboswitches

Among the known purine-based signalling molecules, only c-di-GMP binds to riboswitches in *C. difficile*. C-di-GMP is a signalling molecule that controls the transition from a free-living planktonic lifestyle to a sessile biofilm-associated lifestyle and virulence in bacterial cells [112]. The cyclic dinucleotide initiates signal transduction by binding to protein effectors in most bacteria and to RNA riboswitches in some Gram-positive and Gram-negative species including bacterial pathogens like *V. cholerae*, *B. anthracis* and *C. difficile*. Two distinct types of c-di-GMP riboswitch with distinct structural features (class I, associated with a GEMM RNA motif and class II) that respond differentially to c-di-GMP have been described [91,92]. Instead of controlling c-di-GMP metabolic genes, c-di-GMP riboswitches often reside upstream of genes involved in processes regulated by c-di-GMP, such as motility, adhesion, cell aggregation, biofilm formation and virulence. *C. difficile* primarily responds to c-di-GMP through riboswitches, and the strain 630 encodes 12 predicted class I and 4 predicted class II c-di-GMP riboswitches (Table 1) [13].

**Fig. 5.**
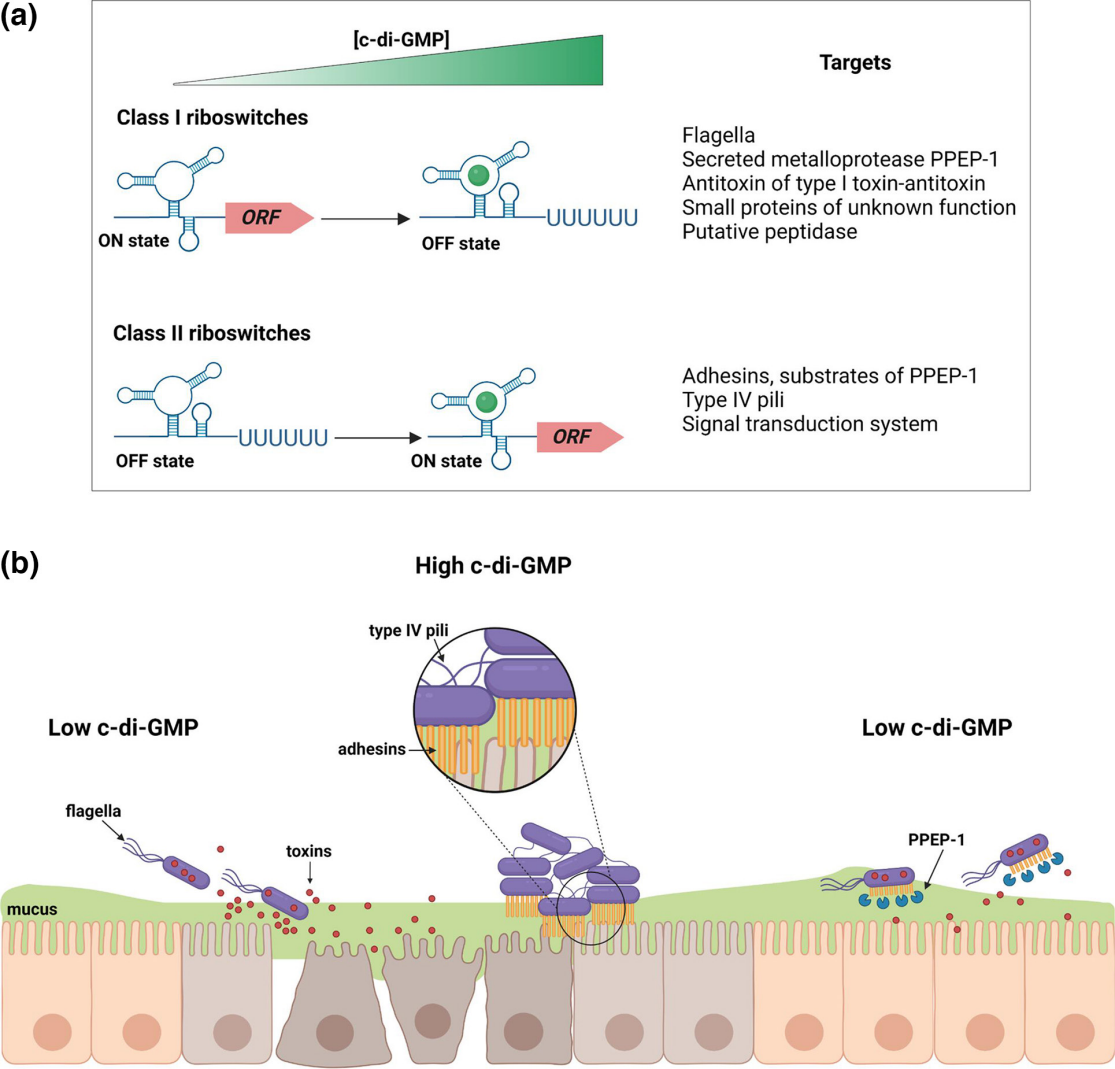
Regulation network of riboswitches responding to c-di-GMP in *C. difficile*. (a) General regulation mechanisms of c-di-GMP riboswitches and controlled pathways. Two types of c-di-GMP riboswitches are present in *C. difficile*. Class I riboswitches act as transcriptional OFF switches, while class II riboswitches are transcriptional ON switches with the exception of riboswitch Cdi2_1, which controls the translation initiation (not depicted). Riboswitches responding to c-di-GMP control the expression of genes in numerous cellular processes including flagellar motility, type IV pilus formation and surface exposure of adhesins. (**b)** Low c-di-GMP concentrations promote swimming motility through the induction of flagellar biosynthesis and toxin production, which could enhance the likelihood of adhesion and propagation of the bacteria in inflamed intestinal tissues. An elevation of the c-di-GMP levels results in a switch from free-living planktonic to a sessile biofilm-associated lifestyle. High c-di-GMP concentrations stimulate type IV pilus formation and production of the CD2831 and CD3246 adhesins to promote cell aggregation, biofilm formation and adherence to the intestinal epithelium. When c-di-GMP concentrations decrease again, the metalloprotease PPEP-1 is secreted and specifically cleaves the two adhesins, resulting in the detachment of *C. difficile* cells from the epithelium. Flagella are concomitantly restored allowing *C. difficile* to spread. Figure created with BioRender.com.

Class I riboswitches act as transcriptional ‘OFF’ switches, while class II riboswitches are transcriptional ‘ON’ switches (Fig. 5a) [113]. Additionally, the class II riboswitch Cdi2_1 controls the translation initiation through c-di-GMP- induced RNA splicing of a group I self-splicing ribozyme to generate a ribosomal-binding site [91,114]. A class I riboswitch controls the expression of the *flgB* operon, which comprises 29 genes encoding early-stage flagella synthesis proteins, and this correlates with the inhibition of swimming motility by c-di-GMP (Fig. 5b) [13,115, 116]. The gene encoding the sigma factor σ^D^ lies within the *flgB* operon, and the expression of the σ^D^ is consequently controlled by the *flgB* riboswitch. Part of the σ^D^ regulon is the gene encoding TcdR, an alternative sigma factor that activates the transcription of the toxin genes *tcdA* and *tcdB* [117]. Consistently, c-di-GMP drastically reduces toxin production in *C. difficile* (Fig. 5b) [118,119]. Another class I riboswitch controls the expression of the Pro-Pro endopeptidase PPEP-1 encoding gene [21]. PPEP-1, also known as ZmpI, is a secreted metalloprotease that cleaves a specific motif comprising a Pro-Pro bond [120,121]. This motif is found multiple times in the C-terminus extremity of two proteins that are covalently anchored to the peptidoglycan of *C. difficile* by the same extremity, and PPEP-1 activity consequently releases these proteins from the cell surface [114,120, 121]. One of the PPEP-1 substrates is a collagen-binding protein, and the other has a thioester-containing domain shown to mediate covalent host binding [120,122]. Notably, the genes encoding these two proteins are controlled by a class II c-di-GMP riboswitch, meaning that PPEP-1 and its substrates are inversely regulated by c-di-GMP [13,114]. As a result, the two adhesins are removed from the cell surface by PPEP-1, and their expression is simultaneously repressed when c-di-GMP concentrations are low (Fig. 5b).

*C. difficile* strain 630 carries two prophages, phiCD630-1 and phiCD630-2, which share a large region of almost identical sequences, including a duplication of a class I c-di-GMP riboswitch [123]. These two riboswitches both control the expression of an ncRNA acting as an antitoxin in a type I toxin–antitoxin system. Antitoxins form a duplex with the toxin mRNA to prevent the translation of the toxin and subsequent growth inhibition. However, these two antitoxins are also expressed from a second, stronger promoter located downstream of the riboswitch sequence, and the riboswitch-associated promoter was shown to be dispensable for toxin inactivation [123]. Other genes controlled by class I c-di-GMP riboswitches encode a putative calcium-binding adhesion protein, a protein containing an Src homology-3 domain and small proteins of 58 aas with no conserved domains [13,113]. In addition to the PPEP-1 substrates encoding genes, class II c-di-GMP riboswitches regulate the expression of a type IV pilus gene cluster and genes encoding the signal transduction system CmrRST. Type IV pili contribute to autoaggregation of *C. difficile* cells and are involved in biofilm formation as well as intestinal colonization [124,126]. CmrRST consists of two response regulators (CmrR and CmrT) and a sensor kinase (CmrS) and plays a critical role in controlling several traits and pathways, including cell and colony morphology, motility, biofilm formation and virulence in an animal model [127,128].

Because c-di-GMP riboswitches control the expression of virulence genes but do not regulate essential metabolic pathways, c-di-GMP analogues targeting these riboswitches are expected to function as antivirulence drugs, preventing infections with no impact on bacterial growth. Circular and linear c-di-GMP analogues binding to a class I riboswitch of *V. cholerae* and to a class II riboswitch of *C. difficile* have been identified [129]. In contrast to the class I aptamer, the class II aptamer interacted strongly with all tested analogues, revealing that it was less selective than the former. Some of these analogues triggered the transcription termination of the class I *flgB* riboswitch or promoted the transcription elongation of a class II riboswitch from *C. difficile in vitro*. However, analogues failed to repress the expression of a reporter gene placed downstream of a class I riboswitch in *E. coli*, raising questions about their functionality *in vivo*.

## Riboswitches binding elemental ions: manganese and fluoride

Although a large proportion of known riboswitches bind and respond to metabolites, several riboswitch classes responding to ions have also been discovered [130]. Riboswitches responding to Mn^2+^ and fluoride (F^−^) are the most broadly distributed in bacteria [131,133]. Less common are riboswitches that recognize Mg^2+^ (Mg^2+^-I and -II riboswitches) [134,135], Li^+^ (Li^+^-I and -II riboswitches) [136], Fe^2+^ (known as NiCo riboswitch as it was first shown to bind Ni^2+^ and Co^2+^ at non-physiological concentrations) [137,138] and Na^+^ [139]. Many of these riboswitches control the genes involved in homeostasis and detoxification of the cognate ion. Only riboswitches binding Mn^2+^ and F^−^ have been identified in the genome of *C. difficile* (Fig. 2a, Table 1).

### Manganese riboswitches

The manganese bivalent cation riboswitches, previously termed *yybP-ykoH* leader RNA element, function as ‘OFF’ switches, modulating the transcription and translation of downstream genes [131,132]. These riboswitches bind Mn^2+^ with high affinity but do not exhibit strict specificity as they also bind other transition metals, including Co^2+^, Cd^2+^ and Ni^2+^ [140]. Mn^2+^ riboswitches are commonly found upstream of genes encoding Mn^2+^ efflux transporters that alleviate cellular Mn toxicity and are, therefore, not considered a suitable drug target [75,141]. *C. difficile* has one predicted Mn^2+^ riboswitch that precedes a gene encoding a putative cation-translocating P-type ATPase. This ATPase is orthologue to YaoB of *Lactococcus lactis*, which is also regulated by a Mn^2+^ riboswitch and was shown to protect the cells against Mn^2+^ toxicity, hinting at a role in Mn^2+^ efflux [132].

### Fluoride riboswitch

Most bacteria are naturally exposed to toxic levels of fluoride, and many species use fluoride riboswitches to regulate the detoxification and adaptation pathways by inducing the transcription or translation when the anion is detected in the surrounding environment [133]. *C. difficile* harbours one fluoride riboswitch upstream of an apparent two-gene operon encoding a putative chloride ion channel (CLC) protein and a protein annotated as a NhaP-type Na^+^/H^+^ or K^+^/H^+^ antiporter. ClC-type proteins that are regulated by fluoride riboswitches are widespread and have been designated EriC^F^. These proteins act as F^−^/H^+^ antiporters that protect the cells from fluoride toxicity by exporting it [133,142].

## Riboswitches binding other metabolites and riboswitch candidates

A few described riboswitches such as the guanidine, the *glmS* and the *yjdF*-binding riboswitches, do not fall into any of the above categories. The guanidyl moiety is a component of larger metabolites such as the nucleobase guanine or the amino acid arginine. Free guanidine is also produced by bacteria, most likely as a toxic byproduct of specific metabolic processes [143]. To counteract the toxicity of guanidine, guanidine riboswitches regulate the genes encoding proteins that are involved in guanidine export and detoxification. The *glmS* riboswitch selectively recognizes the modified sugar compound glucosamine-6-phosphate (GlcN6P) and regulates the expression of the *glmS* gene in numerous Gram-positive bacteria. The GlmS enzyme catalyses the synthesis of glucosamine 6-phosphate (GlcN6P), an essential bacterial cell wall precursor. The *yjdF* riboswitch, found in many Bacillota, binds to an unusually large collection of azaaromatic ligands with only a subset of them activating the gene expression [144]. In addition, other conserved structured RNA motifs believed to be riboswitches but lacking a confirmed ligand have been identified (Table 1).

### Guanidine-I riboswitches

Four distinct structural classes of riboswitches responding to guanidine have been identified in bacteria and are referred to as guanidine-I to guanidine-IV [143,145,148]. The genome of *C. difficile* contains two predicted guanidine-I riboswitches located upstream of genes coding for a SugE-like protein and a B3/4 editing domain-like protein. SugE proteins belong to the small multidrug resistance family, and their genes are commonly associated with guanidine riboswitches. These proteins have been shown to act as guanidine exporters and have been renamed Gdx [149]. The riboswitch preceding *gdx* in *C. difficile* has been shown to specifically induce the transcription upon guanidine binding [143]. Many B3/4-annotated proteins, known as CtdA, are controlled by guanidine-I and -IV riboswitches. This family of proteins prevents the canavanylation of tRNA^Arg^ [143,147]. l-canavanine, or δ-ova-arginine, is a non-proteinogenic amino acid produced exclusively by legume plants and used as the main nitrogen storage compound in seeds. However, canavanine also interferes with arginine metabolism, serving as an antimetabolite of l-arginine [150]. In the presence of canavanine, Arg-tRNA synthetases can mischarge tRNA, resulting in canavanine incorporation into proteins during translation and subsequent protein dysfunction. CdtA proteins have recently been shown to function as editing enzymes, specifically deacylating canavanylated tRNA^Arg^ in legume-associated bacteria as well as in *Clostridium perfringens* [151]. Clostridial species can be exposed to canavanine through the ingestion of canavanine-producing legumes by their animal or human host. The common control of the CdtA encoding gene by a guanidine riboswitch suggests that guanidine, as a degradation product of canavanine, acts as an indicator for canavanine exposure. Because guanidine riboswitches function as ‘ON’ switches, they are considered poor drug targets [143].

### *glmS* riboswitch

*C. difficile*, like many other Bacillota, possesses a single *glmS* riboswitch located upstream of the GlcN6P synthetase gene (*glmS*) [152]. The functionality of this riboswitch in *C. difficile* has recently been validated [153]. The *glmS* riboswitch, often referred to as the *glmS* ribozyme, is mechanistically unique in that it regulates the *gmlS* expression by controlling mRNA stability. It functions as a ribozyme that self-cleaves upon binding to its ligand, GlcN6P, which also serves as a catalytic cofactor for the cleavage reaction [154,155]. This cleavage specifically targets the *glmS* mRNA for intracellular degradation by RNases and consequent reduction of the GlmS enzyme to lower GlcN6P production [156]. As the enzyme is essential, the *glmS* ribozyme is a promising target for antibiotic development. Various GlcN6P mimics, including carba-GlcN6P, have been developed that activate the riboswitch and inhibit bacterial growth [157,161]. In addition, new carba-GlcN6P derivatives with a hydroxy or a methoxy substituent in the carba position were recently shown to induce self-cleavage of the *glmS* ribozyme in *L. monocytogenes* and *C. difficile* [161]. The use of antisense oligonucleotides bound to the cell-penetrating peptide pVEC and targeting the *glmS* ribozyme also led to growth inhibition in *S. aureus* [162].

### yjdF riboswitch

One *yjdF* riboswitch is found in the genome of *C. difficile*. This riboswitch commonly regulates the expression of the YjdF-encoding genes in Bacillota, including *C. difficile*. The function of YjdF is unknown, but this family of proteins harbours a conserved domain of the DUF2992 superfamily. The *yjdF* riboswitch is an ‘ON’ switch binding diverse azaaromatic ligands [144]. A recent study reported that the *yjdF* riboswitch employs an original regulatory mechanism. It mimics the overall shape of the tRNA^Lys^ when bound to activating ligands, and this tRNA-like structure increases the protein expression through direct interaction with the ribosome [163].

### RNA motifs similar to riboswitches

An *uup* RNA motif is located upstream of a gene encoding an ATPase component of ABC transporters of the Uup family in *C. difficile*. Such motifs are found in Bacillota and Gammaproteobacteria and are commonly associated with ATPases with an unknown biochemical function [164]. *uup* RNAs have several highly conserved nts, but their secondary structure is simpler than that of most known riboswitches. There is currently no further evidence that the *uup* RNA motif functions as a riboswitch.

RNA-seq approaches identified a new RNA motif, *bglG*, in *C. difficile* located upstream of the three-gene operon *bglG*, *F* and *A* involved in the uptake and metabolism of aryl-β-glucosides [13,165]. BglG acts as an anti-terminator protein, leading to enhanced transcription of the *bgl* operon [166]. Homologues of the *C. difficile* motif have been identified in Bacillota (RFAM family RF03530) upstream of *bglG* or *licT*, which encodes another antiterminator of a β-glucoside phosphotransferase system [167]. The location and conservation of the *bglG* motif are consistent with a *cis*-regulatory function, but whether this structured motif may function as a riboswitch has not been investigated.

Bioinformatic analysis has identified a single *raiA* RNA motif in the genome of *C. difficile* [164]. This RNA is well represented in the members of Bacillota and Actinomycetota and is often located upstream of a gene encoding the ribosome hibernation factor RaiA [168] or of genes encoding periplasmic-binding proteins, which are transporters of unknown specificity [164]. Additionally, the *raiA* motif is frequently found downstream of *comFC* genes involved in genetic natural competence, although no functional relationship has been established. In *C. difficile*, the *raiA* motif precedes an operon encoding a phosphotransferase system of the lactose/cellobiose family, but the gene directly upstream encodes a ComFC-like protein. Although the *raiA* RNA motif shares some features with riboswitches, it is often located hundreds of nucleotides from the downstream ORF and sometimes in the opposite orientation, which is inconsistent with a function as a riboswitch. Furthermore, a recent study showed that the *raiA* motif operates as a *trans*-acting factor to control the sporulation and cellular aggregation in *Clostridium acetobutylicum* [169].

The *speF* RNA motif is a putative *cis*-acting element found in Gram-negative alphaproteobacteria. This motif is almost always located upstream of the *speF* gene, which encodes an ornithine decarboxylase involved in polyamine biosynthesis [170,171]. No binding of metabolites related to this pathway to the RNA motif could be detected, and there is no strict evidence that the *speF* motif functions as a riboswitch. The presence of a *speF* RNA motif in the genome of *C. difficile* has recently been reported [15]. RNA-seq and Northern blot approaches revealed the presence of a premature transcription termination site associated with the RNA motif, suggestive of a functional riboswitch. However, the gene downstream of the *speF* RNA motif in *C. difficile* encodes a methyltransferase domain protein unrelated to the polyamine biosynthesis pathway. Additionally, the motif in *C. difficile* lacked sequence conservation with described *speF* RNA motif representatives and did not match any RFAM families, suggesting that it might be a different RNA motif.

### T-box riboswitches

**Fig. 6.**
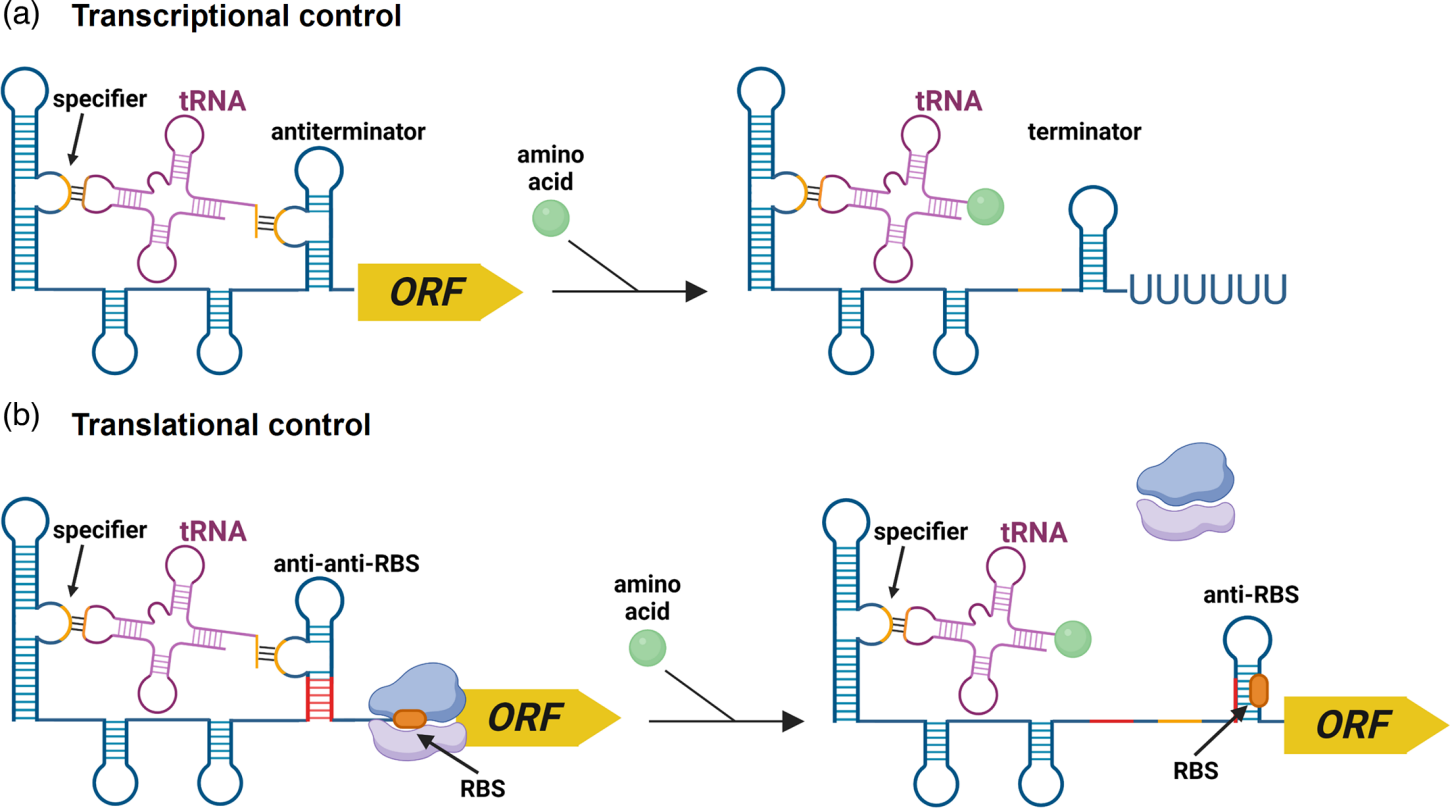
General mechanism of T-box action at the transcriptional or translational levels. (a) Regulation mechanism of T-boxes controlling the transcription termination. Uncharged tRNA binds through base pairing the specifier loop of a cognate T-box and a specific sequence leading to the stabilization of an antiterminator and the transcription of the downstream ORF. Aminoacylated tRNA binds the specifier loop, but the amino acid prevents the binding to the specific sequence, resulting in the formation of a terminator triggering premature termination of the transcription and halting gene expression. (**b)** Regulation mechanism of T-boxes controlling the translation initiation. Uncharged tRNA binds through base pairing the specifier loop of a cognate T-box and a specific sequence leading to the formation of an anti-anti-sequestrator structure, releasing the RBS to allow ribosome binding and translation. Aminoacylated tRNA binds the specifier loop, but the amino acid prevents the binding to the specific sequence, resulting in the sequestration of the RBS, which prevents the translation. Figure created in BioRender.com.

T-box riboswitches are found in Gram-positive bacteria and control the expression of genes encoding aminoacyl tRNA synthetases and proteins involved in amino acid transport and biosynthesis [11] (Table 2). They bind specific tRNAs through a codon–anticodon-like manner and monitor their aminoacylation state as an indicator of amino acid availability to the translating ribosome [11]. T-box binding to a charged or uncharged tRNA will prevent or induce downstream gene expression, respectively, through the control of transcription or translation [172,173] (Fig. 6). There are 25 putative T-box-regulated transcriptional units in *C. difficile* (Table 2, Fig. 2b). Among them, 11 include aminoacyl-tRNA synthetase genes, 9 are amino acid metabolism genes, 4 are amino acid transporter genes and 1 is a gene encoding a protein with an ACT (aspartate kinase-chorismatemutase-tyrA) domain. Proteins with such a domain often interact with amino acids and are involved in some aspects of the regulation of amino acid metabolism [174]. It should be noted that the gene encoding the homoserine dehydrogenase is preceded by two T-boxes, presumably recognizing two different tRNAs.

T-box riboswitches are an attractive drug target as they control the expression of genes that are essential or at least important for competitive fitness in response to amino acid starvation conditions. Chemical compounds, such as aminoglycoside antibiotics and disubstituted oxazolidinone, have been shown to bind the antiterminator RNA in a structure-specific manner and modulate T-box riboswitch activity [175,176]. More recently, small-molecule antibiotics PKZ18 and its analogues have been demonstrated to target the site of the codon–anticodon interaction of T-boxes, reducing downstream gene expression [177,178]. PKZ18 analogues worked synergistically with aminoglycosides and exhibited very low occurrences of drug resistance, making them promising drugs for future clinical application.

## Concluding remarks

Extensive studies over several decades placed the riboswitches at a key position for the regulation of gene expression in bacteria including major pathogens as illustrated in the present review for *C. difficile*. This accumulating knowledge uncovered the diversity of ligands and associated regulatory mechanisms, providing interesting perspectives for future development in biotechnology and health. Riboswitch-derived RNA motifs constitute valuable resources for the design of new synthetic biology modules, molecular sensors and therapeutic strategies.

The decreasing effectiveness of antibiotics against bacterial infections poses a significant public health challenge and a substantial financial burden on healthcare [179]. This issue is further exacerbated by the limited number of new antibiotics in the drug development pipeline [180]. The ESKAPE group of pathogens (*Enterococcus faecium*, *S. aureus*, *Klebsiella pneumoniae*, *Acinetobacter baumannii*, *Pseudomonas aeruginosa* and *Enterobacter* sp.) [181] and *C. difficile* [16] are the primary cause of hospital-acquired infections worldwide. These bacteria are intrinsically resistant to a wide range of antimicrobial agents, making it imperative to identify novel therapeutic targets to fight these infections. Riboswitches may offer a solution, as they possess certain advantages as drug targets. First, many riboswitches control the expression of genes that are essential for bacterial survival or genes that enable the bacteria to succeed in infection. Second, riboswitches are widely distributed throughout the bacterial world but not found in higher eukaryotes, including humans, reducing the risk of adverse effects on human cells. Some riboswitches including those responding to TPP, FMN, SAM and GlcN6P are widespread in bacterial pathogens causing disease in humans and have the potential to be used as targets of broad-spectrum antimicrobials [182]. Conversely, other riboswitches with limited distribution among bacteria could be used to target specific pathogens, thereby preserving the beneficial effects of the protective commensal microbiota. To date, two molecules tested in *C. difficile* have shown encouraging results. The FMN analogue 5FDQD shows bactericidal activity against *C. difficile* with a limited impact on the normal caecal flora of mice when compared to the first-line antibiotics vancomycin and fidaxomicin, used to treat *C. difficile* infections [37]. However, it was not possible to isolate any *C. difficile* mutants that were resistant to 5FDQD, which raises the possibility that the analogue may have multiple targets. Further research is necessary to determine whether the FMN riboswitch is the primary target of 5FDQD. The pyrimidine analogue PC1 is specifically active against bacteria that have a *guaA* riboswitch, including *S. aureus* and *C. difficile* [102]. It is the only analogue to a riboswitch ligand that has been experimentally used as a treatment against *S. aureus*-induced mastitis in cows [183]. However, *S. aureus* could not develop resistance against PC1, suggesting that, as for 5FQD, the molecule may have multiple targets [102]. Since riboswitch ligands are often primary cellular metabolites, targeting other processes than riboswitch conformation with ligand analogues is a major concern, as it could lead to deleterious off-target effects on host cell metabolism. Although compounds targeting a specific riboswitch controlling an essential pathway are the most promising, they also have drawbacks since they can lead to a high frequency of resistance. Mutations will be rapidly acquired in the riboswitch aptamer to disrupt ligand binding to derepress the expression of the essential genes [33]. Targeting several riboswitches of the same class controlling the expression of essential genes with ligand analogue represents a means to solve the resistance issue. For example, resistance to PKZ18 targeting several T-box riboswitches arose at a very low frequency [177]. Nevertheless, the application of this strategy would considerably limit the number of therapeutic candidates. Additional studies are needed to assess resistance development against riboswitch ligands *in vivo*.

## Abbreviations

ABC: ATP binding cassette
AdoCbl: adenosylcobalamin
AEC: L-aminoethylcysteine
AGCS: alanine or glycine:cation symporter
AI2: autoinducer-2
CLC: chloride ion channel
DPD: 4,5-dihydroxy-2,3-pentanedione
ECF: energy-coupling factor
GCV: glycine cleavage
GlcN6P: glucosamine-6-phosphate
GMP: guanosine monophosphate
HyCbl: hydroxocobalamin
LysRS: lysyl-tRNA synthetase
MeCbl: methylcobalamin
ncRNA: non-coding RNA
6-*N*-HAP: 6-*N*-hydroxylaminopurine
PreQ_1_: prequeuosine-1
pVEC: vascular endothelial-cadherin-derived peptide
q: queuine
Q: queuosine
SAM: S-adenosylmethionine
THF: tetrahydrofolate
TPP: thiamine pyrophosphate
5′-UTR: 5′-untranslated region
XMP: xanthine monophosphate
